# Using the circulating proteome to assess type I interferon activity in systemic lupus erythematosus

**DOI:** 10.1038/s41598-020-60563-9

**Published:** 2020-03-10

**Authors:** Michael A. Smith, Chia-Chien Chiang, Kamelia Zerrouki, Saifur Rahman, Wendy I. White, Katie Streicher, William A. Rees, Adam Schiffenbauer, Lisa G. Rider, Frederick W. Miller, Zerai Manna, Sarfaraz Hasni, Mariana J. Kaplan, Richard Siegel, Dominic Sinibaldi, Miguel A. Sanjuan, Kerry A. Casey

**Affiliations:** 1 AstraZeneca, Gaithersburg, MD USA; 2 Environmental Autoimmunity Group, Clinical Research Branch, National Institute of Environmental Health Sciences, National Institutes of Health, Bethesda, MD USA; 3 Lupus Clinical Research Program, National Institute of Arthritis and Musculoskeletal and Skin Diseases, National Institutes of Health, Bethesda, MD USA; 4 Systemic Autoimmunity Branch, National Institute of Arthritis and Musculoskeletal and Skin Disease, National Institutes of Health, Bethesda, MD USA; 5 Immunoregulation Section, Autoimmunity Branch, National Institute of Arthritis and Musculoskeletal and Skin Diseases, National Institutes of Health, Bethesda, MD USA

**Keywords:** Immunology, Biomarkers, Molecular medicine, Rheumatology

## Abstract

Type I interferon (IFN) drives pathology in systemic lupus erythematosus (SLE) and can be tracked via IFN-inducible transcripts in blood. Here, we examined whether measurement of circulating proteins, which enter the bloodstream from inflamed tissues, also offers insight into global IFN activity. Using a novel protocol we generated 1,132 aptamer-based protein measurements from anti-dsDNA^pos^ SLE blood samples and derived an IFN protein signature (IFNPS) that approximates the IFN 21-gene signature (IFNGS). Of 82 patients with SLE, IFNPS was elevated for 89% of IFNGS-high patients (49/55) and 26% of IFNGS-low patients (7/27). IFNGS-high/IFNPS-high patients exhibited activated NK, CD4, and CD8 T cells, while IFNPS-high only patients did not. IFNPS correlated with global disease activity in lymphopenic and non-lymphopenic patients and decreased following type I IFN neutralisation with anifrolumab in the SLE phase IIb study, MUSE. In summary, we developed a protein signature that reflects IFNGS and identifies a new subset of patients with SLE who have IFN activity.

## Introduction

Type I interferon (IFN) is a potent mediator of human immunity. Dysregulation of IFN production or signalling can lead to substantial pathological changes. Demonstrating the centrality of type I IFN in immune responses, exogenous type I IFN administration is sufficient to improve outcomes in patients with chronic viral infection and cancer^1–4^. Additionally, type I IFN neutralisation is an equally promising treatment for autoimmune diseases characterised by unabated type I IFN production^5–8^. At the molecular level, type I IFN induces substantial transcriptional changes in cells, triggering one of the largest cytokine gene networks identified to date^9,10^. IFN-inducible gene signatures have great utility in monitoring IFN activity in humans, revealing links between type I IFN and disease pathology. Type I IFN signatures are strongly linked to disease activity and patient outcomes across diverse indications, including infectious disease, cancer, and autoimmunity^6,11–14^. Thus, the potentially broad translational application of IFN signatures warrants further investigation into additional tools for monitoring the type I IFN pathway in human disease.

Although many translational studies have used IFN-inducible gene scores, there is increasing evidence that analysis of IFN-inducible proteins may provide further insight into human disease. The circulating proteome could complement blood-based gene expression measures by accessing additional biology not reflected by bulk mRNA measurements from circulating cells. Analysis of blood proteins has been used to successfully measure tissue production and deposition of cytokines, complement factors, and hormones, suggesting potential value in disease monitoring when measured with sufficient sensitivity^15–17^. For example, IFN-inducible chemokines were shown to strongly correlate with disease activity and significantly predicted future disease flares in patients with systemic lupus erythematosus (SLE)^18,19^.

Given the valuable information in blood protein profiles, there is increasing interest in measuring proteins with high throughput and sensitivity. DNA aptamer–based assays are a promising technology that offer both^20^. However, these methods have been inaccessible in autoimmune diseases that feature high titres of anti-DNA autoantibodies (eg, anti-dsDNA, anti-ssDNA, and anti-nucleosome antibodies) owing to assay interference. To this end, we evaluated a novel protocol designed to mitigate interference from anti-DNA autoantibodies and, for the first time, we report aptamer-based protein measurements with strong reproducibility and accuracy from sera of patients with SLE. We derived a four-protein score of type I IFN activity (IFNPS) using EPHB2, IP-10, LAG-3, and BLC levels to maximise correlation with an IFN 21-gene signature (IFNGS), a validated pharmacodynamic marker of type I IFN signalling^5,21,22^. We observed a subgroup of patients with high IFNPS who displayed low IFNGS. High-dimensional flow cytometry revealed lower proportions of activated NK, CD4, and CD8 T cells in IFNPS-high/IFNGS-low patients compared with IFNGS-high patients, suggesting that IFNPS can detect IFN activity even when activated cell populations are absent from the blood. IFNPS correlated with global SLE disease activity in both lymphopenic and non-lymphopenic patients, confirming that IFNPS is relatively unaffected by changes in blood cellular composition. Finally, we observed suppression of the type I IFNPS following IFN alpha receptor (IFNAR) neutralisation in MUSE (NCT01438489), a phase IIb study of anifrolumab in moderate to severe SLE, demonstrating specificity of IFNPS for the type I IFN pathway^6^.

## Results

### Anti-DNA autoantibodies interfere with SOMAscan protein measurements

**Figure 1:**
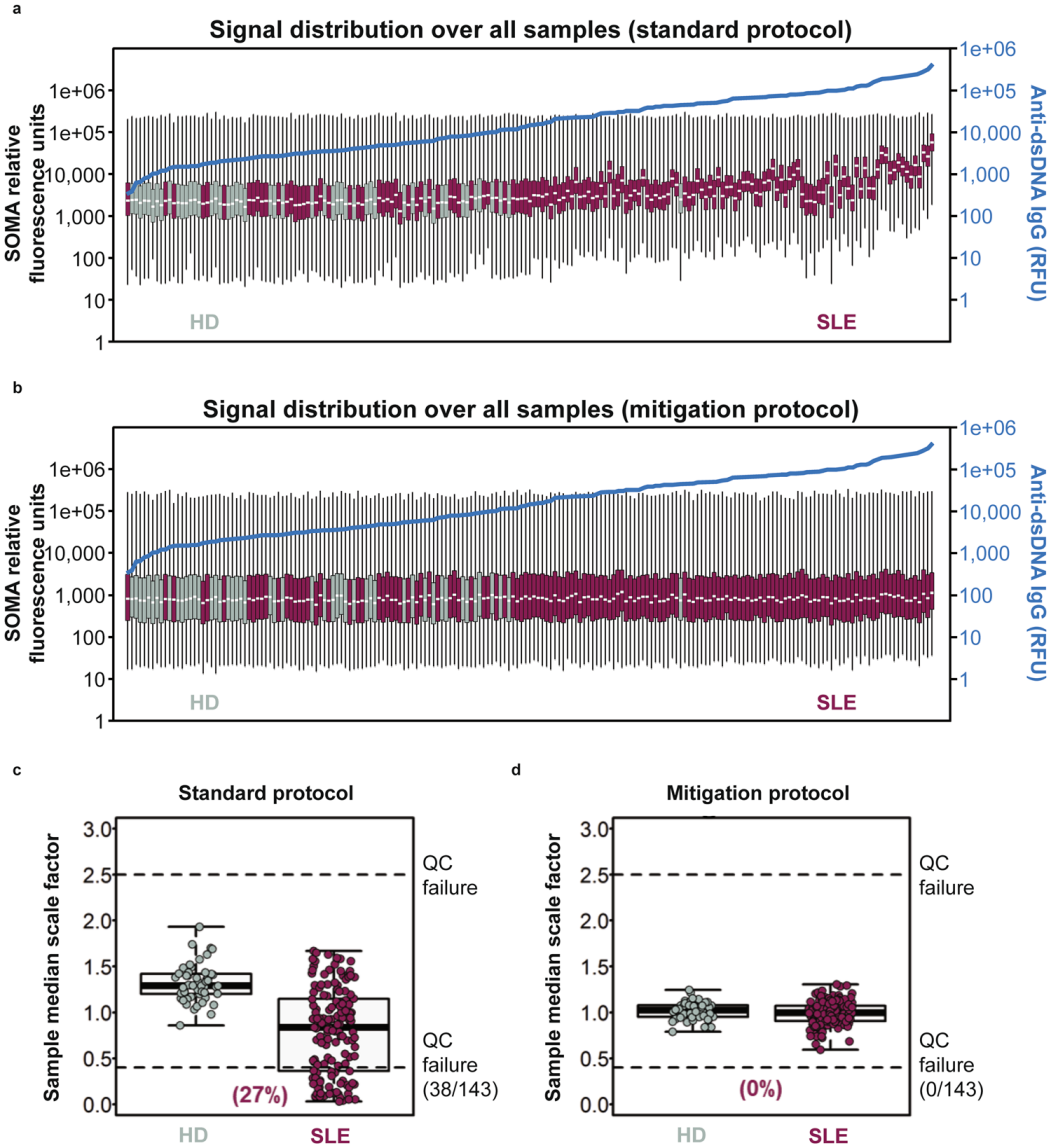
Boxplots displaying global signal distribution of 1,129 protein measurements generated using (**a**) the standard SomaLogic protocol and (**b**) mitigation protocol from serum samples collected from 143 patients with systemic lupus erythematosus (SLE) and 50 healthy donors (HD). The minimum, first quartile, median, third quartile, and maximum relative fluorescence units (RFU) per sample are indicated on each boxplot. Samples are arranged in ascending order based on anti–double-stranded DNA (anti-dsDNA) immunoglobulin G (IgG) RFU. Anti-dsDNA IgG prevalence for each sample is plotted in blue. (**c**,**d**) Sample-specific median scaling factors were used for plate median normalisation in SLE and HD samples. 38 samples from patients with SLE displayed 2.5-fold increased signal relative to their intra-assay control medians, resulting in failure of SOMAscan quality control (QC) standards using the standard SOMAscan protocol. After the samples were processed with the mitigation protocol, all samples passed quality control standards with median signal intensities ranging from a 0.8- to 1.7-fold difference relative to the median assay signal intensity.

DNA aptamer–based protein measurement methods offer unprecedented throughput and sensitivity to quantitate proteins in human disease^23,24^. However, many patients with autoimmune diseases exhibit circulating anti-DNA autoantibodies, which can bind these aptamers and interfere with protein quantitation. We processed serum from 143 patients with mild to moderate SLE and 50 healthy donors (HD). Anti-dsDNA IgG was quantitated by autoantibody array and these continuous measurements were associated with the increased DNA-binding SLEDAI component (Supplementary Fig. S1). We used SOMAscan technology to quantitate 1,129 proteins in each sample. Samples were ordered by anti-dsDNA IgG relative fluorescence units (RFU) after using the standard protocol (Fig. 1a) and the mitigation protocol (Fig. 1b). A total of 38 patients (27%) with SLE displayed 2.5-fold increased signal relative to their intra-assay control medians, resulting in failure of SOMAscan quality control standards (Fig. 1c). Median signal intensity per SOMAscan array strongly correlated with anti-dsDNA autoantibody level in our cohort (Spearman’s r = 0.77, p < 0.0001), consistent with binding between anti-dsDNA autoantibodies and the Slow Off-rate Modified Aptamer (SOMAmer) probes used by the technology.

To mitigate interference of anti-dsDNA autoantibodies with the SOMAmers, we utilised a novel protocol that included adding herring sperm DNA to saturate anti-dsDNA autoantibodies within each sample and prevent these antibodies from binding the SOMAmers. After samples were processed with the new protocol, they passed quality control standards with median signal intensities ranging from a 0.8- to 1.7-fold difference relative to the median assay signal intensity. We found no significant correlation between anti-dsDNA autoantibodies and global signal intensity in measurements once mitigation was applied (Spearman’s r = 0.16, p = 0.06; Fig. 1d). These results indicate that the mitigation protocol prevents anti-dsDNA antibodies from binding SOMAmers, resolving assay interference and enabling aptamer-based protein quantitation in SLE.

### Novel anti-DNA autoantibody mitigation protocol generates protein measurements with high precision and accuracy from sera of patients with SLE

Previous (non-autoimmune or non-SLE) studies have shown that the SOMAscan assay provides measurements with relatively low technical variability and high sensitivity and specificity^23–25^. As such, we investigated whether this performance was retained in measurements generated from the anti-DNA mitigation protocol. We designed an experiment similar to those used to quantify gene expression microarray accuracy and reproducibility and performed experiments in both anti-dsDNA^pos^ and anti-dsDNA^neg^ SLE samples to control for the presence of these autoantibodies^26,27^. We repeatedly assayed three samples to assess reproducibility: one HD, one anti-dsDNA^neg^ SLE, and one anti-dsDNA^pos^ SLE, in triplicate on a single plate (intra-assay precision) and repeated this experiment on three separate days (inter-assay precision). To assess accuracy, we performed serial dilutions of the anti-dsDNA^pos^ and anti-dsDNA^neg^ SLE samples into the HD sample, then measured the ability of the assay to recover the linearity of these dilutions^27^ (Supplementary Fig. S2). We matched both anti-dsDNA^pos^ and anti-dsDNA^neg^ SLE samples for hypocomplementemia, disease severity, and number of analytes with >2 standard deviations of distance from the HD sample (Supplementary Table S1). For each triplicate sample, we observed at least 75% of SOMAscan analytes displayed an intra-assay and inter-assay coefficient of variation (CV) <10% in the mitigated protocol in each sample (Supplementary Fig. S3). In the serial dilutions, we observed more than 80% of analytes with >2 standard deviations from the HD displayed average r^2^ values >0.75 (Supplementary Fig. S4). Together, these results show that SOMAscan measurements generated from the mitigation protocol have high precision and linearity with dilution.

To further assess accuracy of the protein measurements generated from the anti-DNA mitigation protocol, we also examined correlations between paired protein measurements by SOMAscan and Luminex technology SomaLogic, and Rules-Based Medicine (RBM) multiplex immunoassays in 64 anti-dsDNA^pos^ SLE, 79 anti-dsDNA^neg^ SLE, and 50 HD samples. We found that more than two-thirds of SOMAscan measurements from both the standard and anti-DNA mitigation protocols displayed a Spearman’s correlation coefficient >0.5 in HD, anti-dsDNA^neg^ SLE, and anti-dsDNA^pos^ SLE samples (Supplementary Fig. S5). SOMAscan measurements with average Spearman’s correlation coefficient >0.5 and paired Luminex measurements in HD, anti-dsDNA^neg^ SLE, and anti-dsDNA^pos^ SLE samples are listed in Supplementary Table S2. SOMAscan measurements with average Spearman’s correlation coefficient <0.5 in these groups are listed in Supplementary Table S3. In summary, most SOMAscan measurements were reproduced by independent protein assessment technology, further demonstrating specificity of protein measurements generated from the mitigation protocol.

### Development of aptamer-based IFNPS in SLE

Because proteins are secreted by diseased tissues, measurement of IFN-inducible proteins in serum could reveal biological connections between the type I IFN pathway and SLE biology previously unavailable in whole blood gene expression profiles. Furthermore, characterisation of a type I IFN–inducible protein signature could be used to measure IFN activity in patients with SLE in studies that do not have mRNA samples available. Therefore, we sought to derive a protein score of type I IFN activity by identifying a set of proteins that maximises correlation with the IFNGS, a 21-gene score of type I IFN activity^6,21,22,28^. We first selected a training set of 82 SLE and 48 HD samples from our cohort, representing the first sample drawn from each patient. To identify protein measurements that reflect type I IFN activity, we searched for proteins positively and linearly correlated with the IFNGS. We identified a set of 77 SOMAscan measurements associated with the IFNGS with a positive linear correlation >0.3.

**Figure 2:**
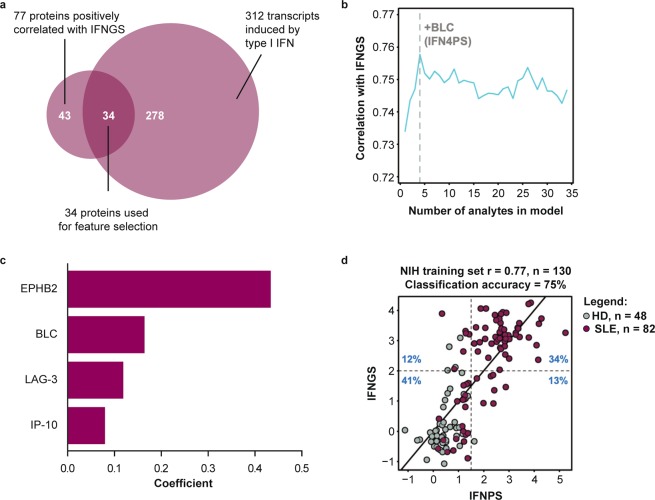
(**a**) Venn diagram displaying selection of protein measurements used for feature selection in LASSO regression. 34 SomaLogic protein measurements displayed a Pearson correlation >0.3 versus the interferon gene signature (IFNGS) and were known to have gene expression inducible by type I interferon (IFN) in human cells. Based on the NIH training set (**b**) average Pearson correlation of IFN protein scores predicted through 10 iterations of 5-fold cross-validation with the IFNGS varying the number of features input into the LASSO regression model. Optimal value of λ was also chosen through 10 iterations of 5-fold cross-validation. Feature selection was restricted to proteins with positive independent associations to the IFNGS. Proteins were scaled to the HD mean and standard deviation prior to fitting the LASSO regression model. (**c**) Regression coefficients from IFN four-protein signature (IFNPS) refit to IFNGS with ordinary least squares regression. (**d**) Scatterplot displaying concordance between IFNPS and the IFNGS. r value calculated using Pearson correlation. SLE = systemic lupus erythematosus, HD = healthy donors.

Type I IFN is a pleiotropic cytokine that induces multiple downstream inflammatory cascades^9,29^. In SLE in particular, sustained type I IFN signalling has the potential to induce vast changes in serum proteins by engaging multiple chronically active inflammatory pathways^30^. As such, proteins correlated with the IFNGS in SLE may be directly induced by type I IFN or indirectly associated with one of these downstream cascades. To identify proteins directly inducible by type I IFN, we determined the intersection of the protein measurements that correlated with the IFNGS with the list of 312 gene transcripts corresponding to SOMAscan protein targets that are transcriptionally induced in human cells upon type I IFN stimulation^31^. The overlap of proteins correlating with the IFNGS and IFN-stimulated genes produced a list of 34 IFN-inducible proteins (Fig. 2a). Using LASSO regression, we identified a set of four proteins, EPHB2, BLC, LAG-3, and IP-10, that optimally correlated with the gene signature. A weighted average of these protein measurements correlated with the gene signature with mean correlation of 0.77 and classification accuracy of 75% after 10 iterations of 5-fold cross-validation in our training set (Fig. 2b–d). In two additional test sets of patients with mild to moderate SLE and HD collected from the same site and independently processed in the years following collection of the training set, this score displayed Pearson’s correlations of 0.68 (p < 0.001) and 0.70 (p < 0.001) with the IFNGS, with classification accuracies of 93% and 77%, respectively (Supplementary Fig. S6). We generated SOMAscan measurements from 305 serum samples taken at baseline during MUSE, a phase IIb study of the safety and efficacy of anifrolumab, which neutralises type I IFN signalling. Using these measurements, we assessed whether the biological pathways and association between type I IFNPS and IFNGS differ across SLE cohorts (ie, patients with mild to moderate SLE and patients with moderate to severe SLE)^6^. In the MUSE cohort, the IFNPS and IFNGS correlated with r = 0.70 (p < 0.001) and displayed a classification accuracy of 78% with the IFNGS (Supplementary Fig. S6). Thus, the selected four-protein IFNPS reflects the IFNGS in multiple SLE cohorts across a spectrum of disease activity, suggesting robust performance of the IFNPS signature in SLE.

Myositis is a systemic autoimmune disease characterised by a high type I IFN–inducible gene signature in some patients that is similar to SLE, but with differing and highly heterogeneous disease manifestations^5,7,32–34^. To understand whether the IFNPS might hold additional promise in the translation to other diseases, we tested whether the IFNPS could still reflect the IFNGS in myositis. Despite extreme clinical heterogeneity of the cohort, with samples from 39 patients with myositis and 65 HD, the IFNPS significantly correlated with the IFNGS, with a Pearson’s r = 0.52 (p < 0.001) and classification accuracy of 84% (Supplementary Fig. S6). This result demonstrates the potential of the IFNPS to reflect type I IFN activity in a disease other than SLE and warrants further investigation in a larger cohort.

A key challenge in the development of any high throughput assay is validation of the specificity of each measurement. We assessed whether SOMAscan measurements of EPHB2, BLC, LAG-3, and IP-10 were independently reproduced by parallel measurements from other platforms to affirm specificity of the four protein constituents of the IFNPS. BLC and IP-10 SOMAscan measurements strongly correlated with matched Luminex protein measurements (Spearman’s r > 0.70, p < 0.001). Although EPHB2 and LAG-3 were not measured by Luminex, we did have paired measurements from the corresponding transcriptional microarray. EPHB2, LAG-3, and IP-10 SOMAscan measurements significantly correlated with gene expression measurements (p < 0.001). BLC displayed low blood gene expression and did not correlate with either Luminex or SOMAscan protein measurement. Each of the four IFNPS measurement components was therefore validated by at least one independent platform supporting the validity of these SOMAmers (Supplementary Fig. S7).

### IFNPS identifies a new subset of patients with evidence of type I IFN activity

**Figure 3:**
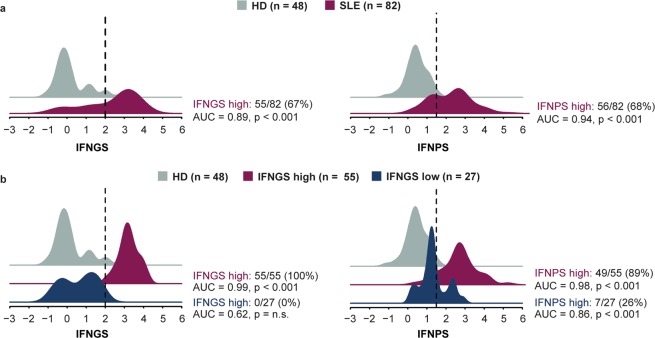
(**a**) Density plots displaying values of interferon gene signature (IFNGS) in systemic lupus erythematosus (SLE; magenta) and healthy donors (HD; grey), and four-protein IFN signature (IFNPS) in HD (grey) and patients with SLE (magenta). (**b**) Values of IFNGS in HD (grey) and patients with SLE with low (blue) and high (magenta) values of IFNGS (cutoff of 2), and IFNPS values in HD (grey) and patients with SLE with low (blue) and high (magenta) IFNGS. Statistical comparisons between each group of patients with SLE and HD were reported with the area under the curve (AUC) and p-value reported from the Mann-Whitney U test.

In patients with SLE, the IFNGS displays a bimodal distribution and can be used to separate patients into two subgroups: those with high IFNGS (IFNGS-high) and those with low levels (IFNGS-low)^21,28^. In the MUSE trial, a four-gene IFNGS test was used to stratify patients, and significant treatment responses in patients with SLE receiving anifrolumab versus placebo were primarily observed in IFNGS test–high patients^6^. Although IFNGS test–low patients treated with anifrolumab displayed less prominent treatment responses versus placebo than IFNGS test–high patients, improvements in rash and arthritis were observed in this cohort, suggesting that some IFNGS test–low patients might have pathology that induces type I IFN activity despite a low IFNGS test score^35^. Serum proteins can originate from SLE-affected tissue compartments, whereas blood IFN-inducible genes are averaged from cells circulating in the blood; therefore, we hypothesised that the IFNPS might detect additional IFN activity in some IFNGS-low patients. We compared prevalence of the IFNPS in HD with both IFNGS-high and IFNGS-low patients with SLE and found that IFNPS was strongly elevated in all patients with SLE. Of patients with SLE, 68% displayed an IFNPS >2 standard deviations from the HD mean (AUC = 0.94, p < 0.001). The IFNPS was also significantly elevated in IFNGS-low patients with SLE (AUC = 0.86, p < 0.001), and a subset of 26% of IFNGS-low patients with SLE who similarly displayed an IFNPS >2 standard deviations from the HD mean was identified (Fig. 3). This result provides evidence that the IFNPS can be used to identify a unique subset of patients with potentially high type I IFN activity and low levels of IFN-inducible genes in the blood.

**Figure 4:**
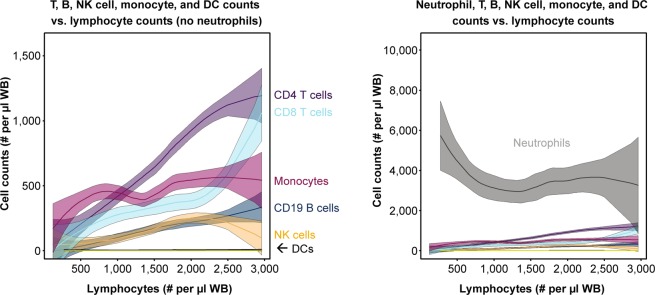
Line plots displaying relationship between cell counts and lymphocyte counts. Line represents best fit from local regression with degree 2 and span 0.75. Shaded region represents a 95% confidence interval of the mean. DC = dendritic cells, NK = natural killer cells.

**Figure 5:**
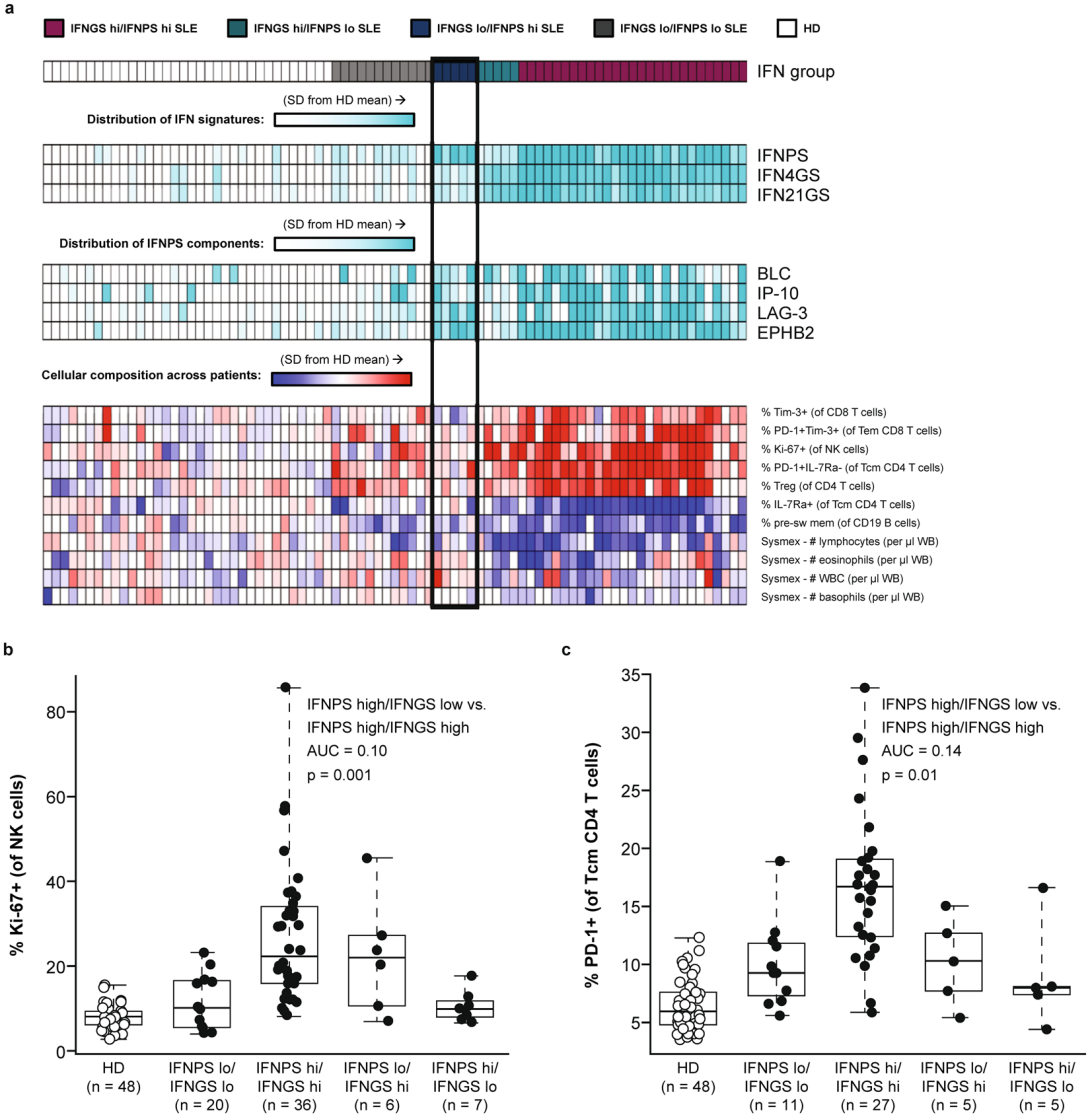
(**a**) Heatmap displaying associations of cell populations with interferon gene signature (IFNGS) and interferon protein signature (IFNPS) in 3-way ANOVA (p < 0.05). (**b**,**c**) Boxplots displaying elevation of Ki-67+ NK cells and PD-1+ Tcm CD4 T cells in IFNGS-high patients with systemic lupus erythematosus (SLE) versus IFNPS-high/IFNGS-low patients with SLE. AUC = area under the curve, HD = healthy donors, SD = standard deviation, WB = whole blood. NK = natural killer cells, Tcm = central memory T cells, Tem = effector memory cell.

Blood gene expression profiles are derived from the cellular composition of blood samples. SLE is characterised by large compositional variation between patients, shown in our cohort as a function of lymphopenia in Fig. 4. Neutralisation of type I IFN signalling has been shown to correct SLE-associated dysregulation of lymphocyte, neutrophil, monocyte, and platelet numbers in the blood, suggesting a role for type I IFN in the establishment of this heterogeneity^36^. Given the circulating half-life of proteins and their ability to filter into the blood from inflamed tissues, a protein signature might be insensitive to compositional change. Therefore, we set out to identify key cell populations associated with IFN signatures and measured the association between IFNPS, IFNGS, and prevalence of different cell populations with multicolour flow cytometry. Using multiple linear regression models that measured the association of both the IFNPS and IFNGS with each cell population, we identified 13 blood compositional changes uniquely associated with the IFNGS. Ki-67^+^ NK cells, PD-1^+^ and Tim-3^+^ T effector memory CD8 T cell populations, and PD-1^+^ IL-7Ra^−^ CD4 T cells were found to be significantly elevated in IFNGS-high patients and were not elevated in IFNPS-high/IFNGS-low patients (Fig. 5, Supplementary Table S4). This result suggests that activated NK, CD4, and CD8 T cell populations could be fundamental contributors to the blood IFNGS and that the IFNPS could identify some patients with high type I IFN activity who do not display activation of these cell populations. The independence of IFNPS from key compositional changes further suggests that the IFNPS could be produced by affected SLE tissues, not within the blood.

### IFNPS and IFNGS correlate with global disease activity in SLE

**Figure 6:**
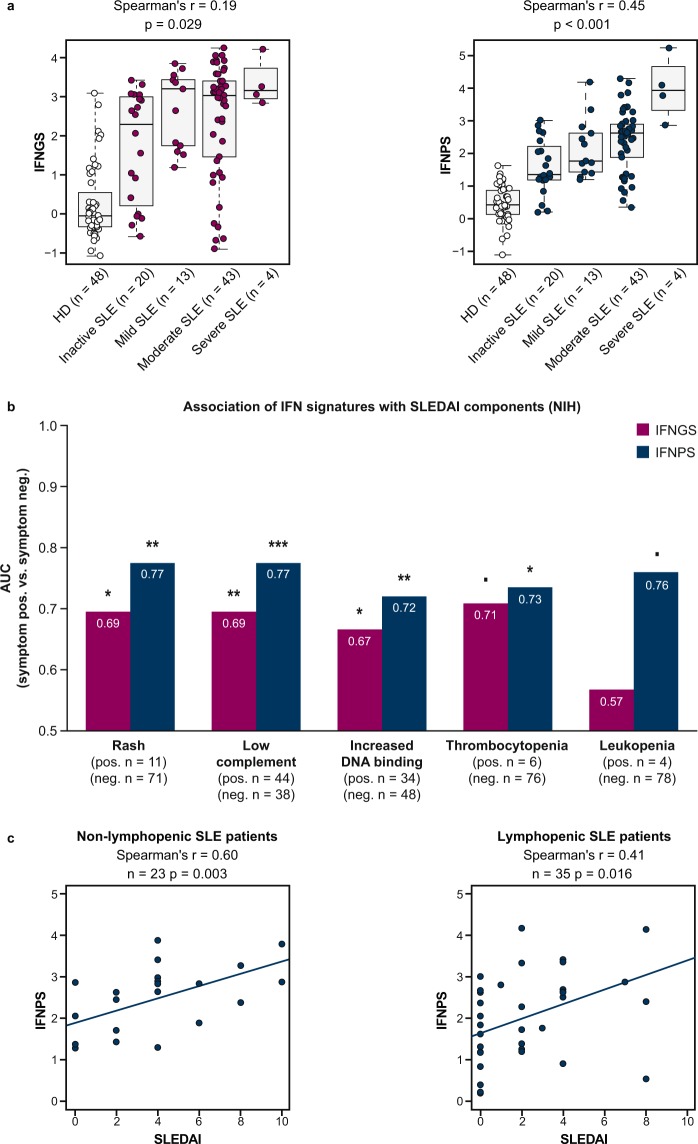
(**a**) Boxplots displaying correlation between interferon gene signature (IFNGS) and interferon protein signature (IFNPS) with systemic lupus erythematosus (SLE) disease activity. (**b**) Area under the curve (AUC) of IFNGS and IFNPS in discriminating patients with SLE positive or negative for specific SLE Disease Activity Index (SLEDAI) components in the NIH lupus cohort. Threshold for leukopenia: <3,000 WBC/µl. (**c**) Scatterplots displaying correlation between IFNPS and SLEDAI in non-lymphopenic patients with SLE and lymphopenic patients with SLE. P-values reported using Mann-Whitney U test (***p < 0.001, **p < 0.01, *p < 0.05, ▪p < 0.10). HD = healthy donors.

Because the IFNPS detected signals in patients with strong evidence of immune cell activation in the blood, we further characterised the association between the IFNPS and composite disease activity in the training set to understand if, as previously demonstrated for the IFNGS, the IFNPS correlates with overall disease activity. We calculated the Spearman’s correlation of the IFNPS and IFNGS with the SLE Disease Activity Index (SLEDAI), an assessment of SLE disease activity across multiple organ systems^37^. We found that IFNPS shared a Spearman’s correlation of 0.45 (p < 0.001) with SLEDAI, while the IFNGS displayed a Spearman’s correlation of 0.19 (p = 0.029) with SLEDAI, suggesting the IFNPS could serve as a useful biomarker of composite disease in SLE (Fig. 6a).

We then sought to understand which individual components of the SLEDAI composite score drive this association. We examined prevalence of the IFNPS and IFNGS in patients positive and negative for each SLEDAI component. Both the IFNGS and IFNPS were significantly elevated in patients who presented with rash, low complement, and anti-dsDNA autoantibodies. The IFNPS was also significantly elevated in thrombocytopenic patients with SLE, and the IFNGS displayed a similar trend. The IFNPS also displayed numerical elevation in leukopenic patients with SLE (Fig. 6b). We further verified that the IFNPS significantly correlates with SLEDAI in both lymphopenic and non-lymphopenic patients with SLE (p < 0.05), providing further evidence that the signature might reflect tissue biology that is insensitive to blood compositional changes (Fig. 6c). SLEDAI component and summary scores, anti-dsDNA IgG RFU measurements, and IFNPS and IFNGS scores for each patient are provided in the Supplementary Data.

To determine whether these findings could be reproduced in an independent cohort of patients with moderate to severe disease, we validated the association between the IFNPS and SLEDAI composite score in both lymphopenic and non-lymphopenic patients in the MUSE cohort. We also found significant association between IFNPS and hypocomplementemia, increased anti-dsDNA, and leukopenia in this cohort (Supplementary Fig. S8). Only two thrombocytopenic patients with SLE were recruited in the MUSE cohort; thus, we could not validate the association between IFNPS or IFNGS and this SLEDAI component. In this cohort, we observed that the IFNPS was not significantly associated with the SLEDAI rash component but did display a positive correlation with Cutaneous Lupus Erythematosus Disease Area and Severity Index (CLASI) activity score (Spearman’s r = 0.21, p < 0.001), an alternative measure of cutaneous disease activity, confirming the association between IFNPS and SLE cutaneous involvement^38^. In summary, we found that the IFNPS reflects inflammation across multiple organ systems in patients with SLE, making the IFNPS a useful biomarker of composite disease activity.

### IFNPS is associated with the type I IFN pathway

Type I and type II IFNs have distinct roles in amplifying immune response but induce largely overlapping transcriptional changes in cells^39^. Moreover, type II IFNs are directly inducible by type I IFNs^40^. For these reasons, distinguishing between both types of responses while monitoring human disease can be challenging. To verify that the IFNPS we identified is reflecting type I IFN– and not type II IFN–associated biology, we first measured the correlation between IFNPS and transcription of several components of IFN-γ–inducible gene signatures, IRF1, CXCL9, and SLAMF8^39,41^, and found no correlation between the IFNPS and these genes in samples from patients with either SLE or myositis. In contrast, the IFNPS correlated with all four components of a type I IFN–inducible gene signature, IFI44L, IFI27, RSAD2, and IFI44, providing evidence that the IFNPS is directly induced by type I IFNs and not type II IFNs^6^ (Supplementary Fig. S9).

**Figure 7:**
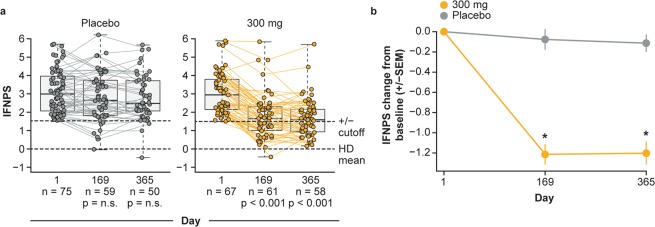
(**a**) Interferon protein signature (IFNPS) over time in patients who received placebo or anifrolumab 300 mg in the MUSE study. Box and whiskers represent patient quartiles. (**b**) Mean change from baseline in interferon protein signature over time. Error bars represent standard error of the mean (SEM). *p < 0.05 by Mann-Whitney U test. HD = healthy donors, n.s. = not significant.

To further evaluate whether the IFNPS is specifically induced by type I IFNs, we investigated whether the IFNPS is suppressed by neutralisation of IFNAR, the receptor necessary for type I IFN signalling. We monitored the IFNPS of patients with SLE before and after treatment with anifrolumab, a monoclonal antibody that neutralises IFNAR, in the MUSE trial^6^. We found that the IFNPS was significantly decreased at Days 169 and 365 compared with Day 1 (p < 0.001) in the anifrolumab 300-mg treatment group. In contrast, the IFNPS displayed no significant changes from baseline in the placebo group (p > 0.05; Fig. 7a). Changes in the IFNPS from Day 1 to Days 169 and 365 were also significant when compared between the anifrolumab 300-mg group and placebo group (Fig. 7b). Therefore, the IFNPS was specifically suppressed following IFNAR neutralisation with anifrolumab, further indicating that the IFNPS reflects biology induced by type I IFN.

## Discussion

We utilised a novel protocol that included adding herring sperm DNA to saturate anti-dsDNA autoantibodies (to mitigate interference of anti-dsDNA autoantibodies with SOMAmers) to measure aptamer-based proteins generated from SLE sera with high throughput, precision, and accuracy. We used these measurements to generate a novel type I IFNPS and identify a new subset of patients with type I IFN activity in SLE and myositis. This protein signature could be applied to immune monitoring in autoimmune diseases such as SLE, in which type I IFNs are dysregulated, as well as other inflammatory conditions including tuberculosis infection or cancer immunotherapy, for which understanding the role of type I IFN in pathobiology is paramount.

Differences between the IFNPS and type I IFN–inducible gene signatures could be explained by the fact that serum proteins can originate from multiple tissues, whereas transcriptional signatures are solely dependent on the cellular composition of blood. In support of this hypothesis, we found the IFNPS to be insensitive to blood composition changes that occur in IFNGS-high patients with SLE, suggesting that the IFNPS could be produced not just from blood cells, but also from the disease-affected tissues of patients with SLE. In further support of this hypothesis, we found that serum BLC measurements did not correlate with blood-derived gene expression of BLC, whereas we would expect blood gene expression and blood protein prevalence to correlate if BLC was primarily produced in the blood. Plasma prevalence of BLC has recently been shown to strongly correlate with the germinal center frequency of T follicular helper cells after vaccination, indicating that tissue resident cells are the main drivers of serum prevalence of this chemokine^42^. In contrast, EPHB2, IP-10, and LAG-3 protein measurements significantly correlated with gene expression measurements, suggesting that these proteins could be produced in part by blood cells. Because the IFNPS could be produced outside of the blood, at least in part, this signature could be useful in monitoring type I IFN in conditions in which pathology occurs in tissues, such as SLE. However, further work is needed to determine where these individual proteins are produced. Additionally, investigations in subsets of patients with SLE who do not have elevated IFNGS or IFNPS, as well as patients with nephritis, for example, would be informative.

A limitation of this study is that we have not had the opportunity to measure type I IFN–inducible proteins in other inflammatory diseases outside of SLE and myositis. It would still need to be determined whether the protein signature we have identified also reflects type I IFN in broader contexts such as cancer and chronic infections. Extended-duration interventional studies with targeted inhibitors of the type I IFN pathway will be especially informative. It is also unknown whether the identified IFN protein signature would identify biology unique from IFN-inducible gene scores in these contexts. In SLE, type I IFN signalling is tightly linked to numerous compositional changes in blood cell populations^36^. As such, there might be an even stronger agreement between the IFNPS and IFNGS in other conditions in which blood composition is less varied across patients compared with SLE.

In conclusion, our results suggest that type I IFN–inducible proteins have the potential to detect unique tissue biology not available in blood gene expression measurements. Because type I IFN is a central regulator of immune response, this signature may have application in understanding the pathobiology of multiple human diseases. In multi-organ diseases, such as SLE, this signature could prove essential to fully understanding the role of type I IFN.

## Methods

### Patients and sample collection

A total of 143 whole blood samples from patients with SLE were obtained from the NIH Clinical Center in Bethesda, MD, USA, and shipped to MedImmune LLC, previously a subsidiary of AstraZeneca, in Gaithersburg, MD, USA, on a weekly basis throughout 2014 under clinical protocol NIH 94-AR-0066, approved by the National Institute of Arthritis and Musculoskeletal and Skin Diseases/National Institute of Diabetes and Digestive and Kidney Diseases (NIAMS/NIDDK) Institutional Review Board (IRB). A total of 50 whole blood samples from HD were also obtained on a weekly basis under the internal donor program, approved by the MedImmune IRB, processed, and stored simultaneously with SLE samples arriving from the NIH. In 2015, an additional test set of seven SLE samples and eight HD samples were collected from the NIH and MedImmune, respectively, under the same protocols. In 2016 and 2017, an additional test set of samples from 68 patients with SLE and 55 HD were collected from the NIH and MedImmune, respectively, under the same protocols. Samples from patients with myositis were collected under protocol NIH 94-E-0165, approved by the same IRB, with collection occurring between 2015–2017. Patient and HD clinical characteristics are listed in Supplementary Table S5.

For samples from the NIH cohort, whole blood was collected by peripheral venipuncture and serum was isolated using serum-separator Vacutainer tubes (Becton-Dickinson catalog #367988, Palo Alto, CA, USA). Aliquots were stored immediately at −80 °C. Blood was prepared for whole blood gene expression profiles and flow cytometry using the PAXgene system (Becton-Dickinson catalog #762125, Palo Alto, CA, USA).

A further test set of 305 SLE serum samples was collected from a pre-dose time point in MUSE (NCT01438489), a randomised, double-blind, placebo-controlled phase II clinical trial investigating the efficacy of anifrolumab, an anti–type I IFNAR subunit 1 antibody, for the treatment of SLE. The detailed trial design, collection of serum samples, and measurement of the IFNGS in this cohort have been previously described^6,43^. All patients provided informed consent to participate in the study. No personal identification was provided for the analysis.

A total of 10 serum samples were purchased from Bioreclamation IVT, aliquoted, and used as bridging samples for the 2014 NIH training set, the 2015 and 2016–2017 NIH test sets, and the NCT01438489 test set. These samples consisted of six HD, one patient with chronic obstructive pulmonary disease, one patient with rheumatoid arthritis, one patient with SLE, and one patient with Crohn’s disease. Aliquots of these samples were shipped simultaneously to SomaLogic and RBM along with each of the three sample sets. SLE samples from MUSE were normalised to the six purchased HD samples for calculation of the IFNPS.

### Serum protein measurements

In the 2014 training set, 1,129 proteins were measured in serum from each sample using affinity-purified, modified DNA aptamer probes, which were subsequently quantified with a custom Agilent hybridisation chip as described using the SOMAscan Assay v3.2 SLE-mitigated protocol and SOMAscan Assay v3.2 standard protocol (SomaLogic, Boulder, CO, USA)^20,44^. Protein measurements from the 2015 and 2016–2017 NIH lupus test sets and the MUSE test set were generated using only the SOMAscan Assay v3.2 SLE-mitigated protocol. A total of 179 proteins were also measured in the 2014 NIH lupus cohort serum samples by Luminex immunoassay using the Human DiscoveryMAP® 175+ v. 1.0 (Myriad RBM, Austin, TX, USA).

Serum anti-dsDNA IgG RFUs were measured as a component of the UTSW autoantibody array for validation of the mitigation protocol. Clinical ELISAs were also used to measure serum anti-dsDNA at the time of clinic visit, as a component of SLEDAI. As anti-dsDNA IgG assays may vary in sensitivity and specificity in patients with SLE^45^, measurements obtained from the UTSW array are shown for patients with and without increased DNA binding and full patient-level details have been provided in the supplement (Supplementary Fig. S1 and Supplementary Data).

### Gene expression microarray and calculation of IFN 21-gene signature

Whole blood gene expression microarray measurements were performed using the Affymetrix Human Genome U133 Plus 2.0 GeneChip array platform. Sample processing and array hybridisation was performed by Asuragen (Austin, TX, USA), and the IFN 21-gene signature (IFNGS) was calculated, as described previously^21^.

### Flow cytometry surface and intracellular staining

One million freshly isolated peripheral blood mononuclear cells (PBMCs) from HD and SLE blood samples were used for flow cytometry. Fc receptor blocking (TrueStain FcX, Biolegend) was performed in PBMCs for 15 minutes in staining media (PBS with 1% BSA and 0.1% sodium azide) on ice. Cells were then stained with antibody mix for 30 minutes, washed twice with staining media, and fixed in freshly prepared 4% paraformaldehyde. Intracellular staining for Ki-67 was performed using permeabilization buffer as per manufacturer’s instructions (eBioscience, USA). The fixed cells were then acquired on multicolour flow cytometry instrument BD LSRFortessa (BD Biosciences, USA), and data were analysed using FlowJo software (BD Biosciences, USA). Cell number calculations as well as lymphocyte and granulocyte population enumerations were performed using complete blood count haematology analyser (Sysmex, Japan).

The following antibodies were used: CD4 (BD Biosciences, clone: RPA-T4), CD8a (BD Biosciences, clone: RPA-T8), CD19 (BioLegend, clone: HIB19), CD27 (BioLegend, clone: O323), CD45RA (BD Biosciences, clone: HI100), CD56 (BD Biosciences, clone: NCAM16.2), CCR6 (BD Biosciences, clone: 11A9), CCR7 (BioLegend, clone: G043H7), CXCR3 (BD Biosciences, clone: 1C6/CXCR3), IgD (BioLegend, clone: IA6-2), Ki-67 (BD Biosciences, clone: B56), IL-7Ra (BD Biosciences, clone: HIL-RA-M21), PD-1 (BD Biosciences, clone: EH12.1), TCRab (BioLegend, clone: IP26), and Tim-3 (R&D Systems, clone: 344823).

### SOMAscan precision and accuracy

To assess technical variation of SOMAscan measurements, samples from HD, patients with anti-dsDNA^neg^ SLE, and patients with anti-dsDNA^pos^ SLE were assayed in triplicate repeatedly on three separate days. Volumetric titrations between samples were also performed between each selected patient with SLE and HD at 75:25, 50:50, and 25:75 ratios to assess linearity of measurements with titration. These measurements were generated for both the SOMAscan standard and mitigation protocols.

### Statistical analysis

All statistical analyses were conducted in R 3.1.1. Pairwise correlations were calculated using the non-parametric Spearman’s correlation unless otherwise stated. Pairwise comparisons were calculated using the non-parametric Mann-Whitney U test.

CV was calculated per analyte between three replicate measurements assayed on the same multi-well plate for anti-dsDNA^pos^, anti-dsDNA^neg^, and HD samples to assess intra-assay variation, and between three replicates processed and run on separate dates to assess inter-assay variation.

To compare linearity of SOMAscan measurements from the mixed dilutions in both patients with anti-dsDNA^pos^ and anti-dsDNA^neg^ SLE, the r^2^ value was calculated for an ordinary least squares (OLS) fit. The relative fluorescent units reported for the technical replicates of each SLE sample, HD, and the three mixed dilutions run within the same multi-well plate were used as the dependent variable. The mixing ratios between samples were used as the independent variable.

To identify a protein score that predicts the IFN 21-gene signature (IFNGS), we first reduced our set of 1,129 protein measurements from the 2014 NIH lupus training set to a set of 33 proteins known to be induced by type I IFN in human cells as listed by the INTERFEROME database with significant positive correlation to the IFNGS (Spearman’s r >0.3)^31^. Prior to feature selection, all protein measurements were log_2_ transformed and scaled to the mean and standard deviation of the 2014 HD distribution for each protein. We then used LASSO regression for feature selection. The shrinkage parameter, λ, and the number of top correlates of the IFNGS, *k*, to include in the model were chosen based on the values that minimised mean squared error with the IFNGS and LASSO regression predictions after 10 iterations of 5-fold cross-validation. The R *glmnet* package was used to fit the LASSO model. A linear combination of the top four protein correlates of the IFNGS optimally predicted the IFNGS in the training set. We refitted the model composed of the four protein measurements using OLS regression to derive final coefficient estimates.

In the two NIH test sets, the four protein measurements were log_2_ transformed and scaled to the mean and standard deviation of the respective HD distribution prior to calculation of the IFNPS. In the MUSE test set, the four protein measurements were log_2_ transformed and scaled to the mean and standard deviation of six HD bridging samples. These bridging samples were also repeatedly assayed across the NIH 2014, 2015, and 2016–2017 cohorts and were found to approximate the HD distribution for each of the four IFNPS components.

To identify cell populations associated with the IFNGS and IFNPS, a multiple regression model was fitted using each cell population sequentially as an independent variable, treating IFNGS-high/-low, IFNPS-high/-low, and disease status (SLE vs. HD) as covariates. An F-test was used to assess statistically significant associations between different cell populations and the IFNPS and IFNGS combined. Benjamini and Hochberg FDR correction was applied to p-values from this F-test, and cell populations were significant with FDR < 0.10. Post hoc testing was then applied to assess the independent association of either the IFNGS or IFNPS with each cell population, and p-values < 0.05 were considered significant.

To assess changes of the IFNPS with anifrolumab treatment, a Wilcoxon signed-rank test was first used to assess whether the IFNPS changed significantly from baseline in either the placebo or anifrolumab 300-mg group. A Mann-Whitney U test was then used to compare baseline subtracted IFNPS at Days 169 and 365 to assess whether these changes from baseline were significant between treatment groups.

### Compliance with ethical standards

All methods were carried out in accordance with relevant guidelines and regulations. All experiment protocols were approved by the relevant IRB. Samples from patients with SLE and myositis were collected from the NIH under clinical protocols NIH 94-AR-0066 and NIH 94-E-0165, respectively, which were approved by the NIAMS/NIDDK IRB. Samples from HD were collected by MedImmune under the internal donor program as approved by the MedImmune IRB. All patients provided informed consent to participate in the study.

## Supplementary information

Dataset 1.

Supplementary Information.

## Data Availability

Data underlying the findings described in this manuscript may be obtained in accordance with AstraZeneca’s data sharing policy described at https://astrazenecagrouptrials.pharmacm.com/ST/Submission/Disclosure.
