# Supplementary material for: Using the circulating proteome to assess type I interferon activity in systemic lupus erythematosus
Source: Sci Rep. 2020 Mar 10;10:4462. doi: 10.1038/s41598-020-60563-9 (PMC7064569; doi:10.1038/s41598-020-60563-9)
Supplement: Supplementary file 2 — Supplementary Information. [file 41598_2020_60563_MOESM2_ESM.docx]

**SUPPLEMENTARY INFORMATION**

**Using the Circulating Proteome to Assess Type I Interferon Activity in Systemic Lupus Erythematosus**

*Michael A. Smith, Chia-Chien Chiang,* *Kamelia Zerrouki, Saifur Rahman, Wendy I. White,
Katie Streicher,* *William A. Rees, Adam Schiffenbauer, Lisa G. Rider*, *Frederick W. Miller,
Zerai Manna, Sarfaraz Hasni, Mariana J. Kaplan, Richard Siegel,* *Dominic Sinibaldi,
Miguel A. Sanjuan,* *Kerry A. Casey*


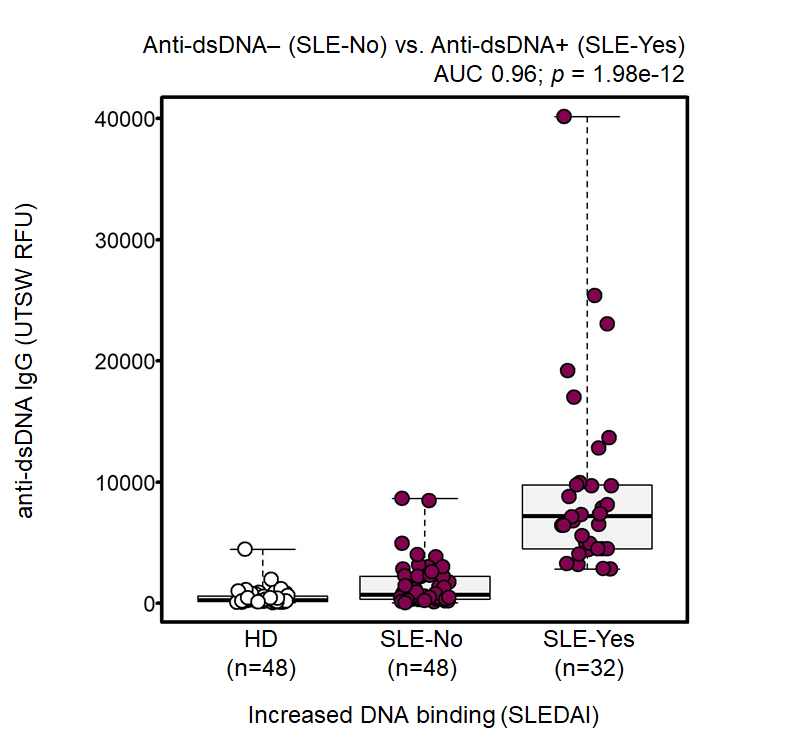


Supplementary Figure S1. Serum anti-dsDNA IgG measured using the UTSW autoantibody array in patients with SLE who have positive or negative SLEDAI-increased DNA-binding test results (SLE-Yes or SLE-No) and in healthy donors. Box and whiskers represent quartiles of each group.


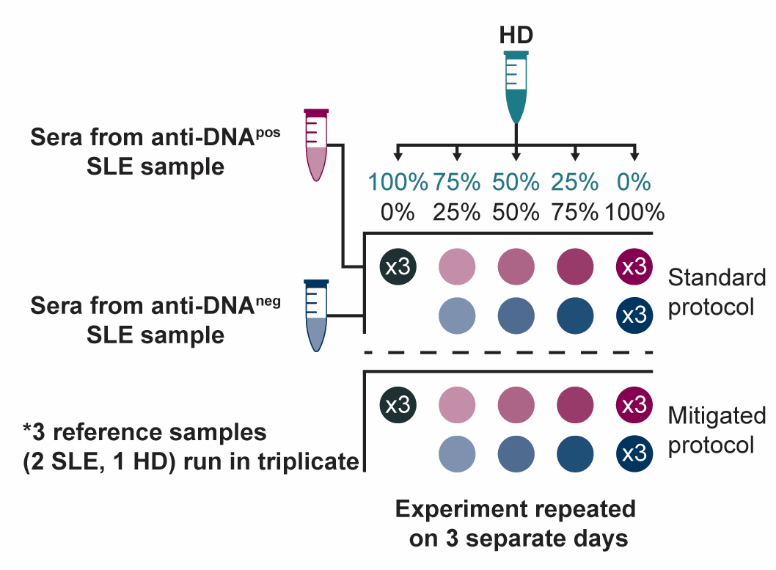


Supplementary Figure S2. Mixed dilution study design. Similar to MAQC-I and MAQC-III studies, serum from an anti–double-stranded DNA (anti-dsDNA)^pos^ systemic lupus erythematosus (SLE) sample, an anti-dsDNA^neg^ SLE sample, and a healthy donor (HD) sample were analysed in triplicate to assess reproducibility of SomaLogic measurements. SLE samples were then titrated into the HD sample with known 3:1, 1:1, and 1:3 ratios to assess ability of technology to recover known mixing ratios. This experiment was then repeated using the SomaLogic standard and anti-dsDNA mitigation protocol on three separate days.


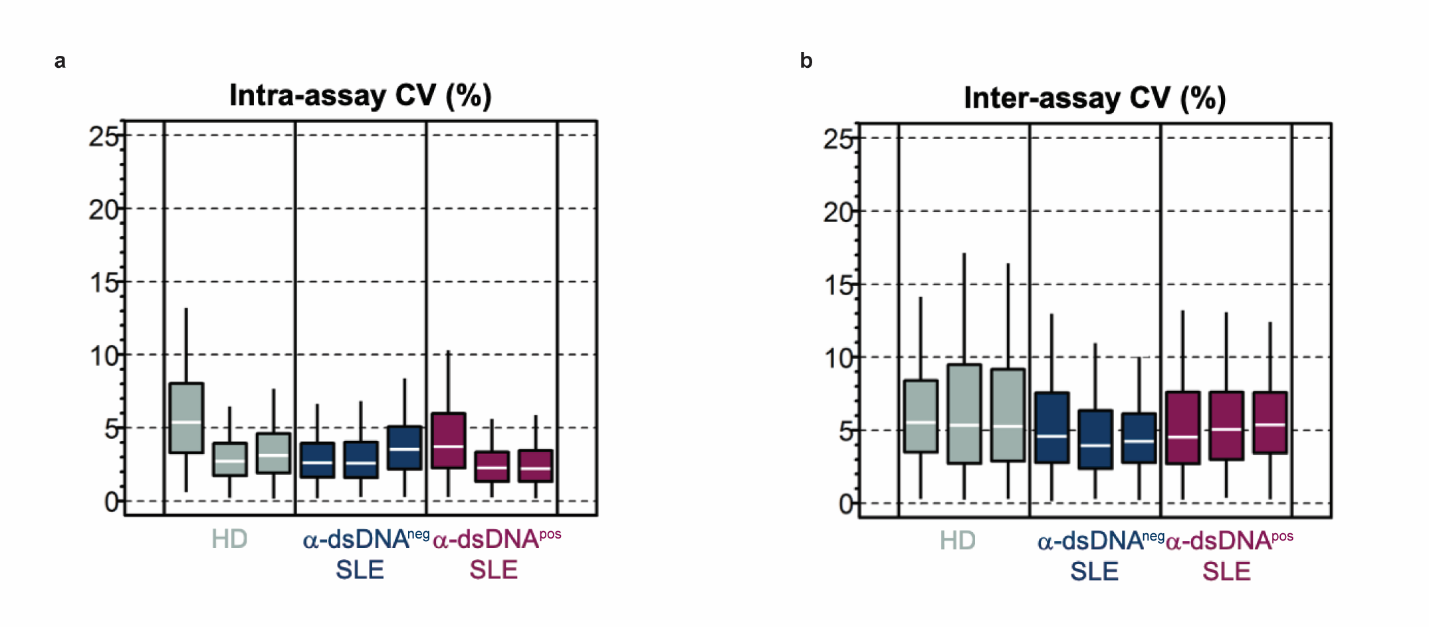


Supplementary Figure S3. Reproducibility of relative fluorescence units (RFU) of samples run (a) within same plate on same day and (b) on different plates on different days under the mitigation protocol. Boxplots represent the 10th and 90th percentile, interquartile range, and median distribution of coefficient of variation (CV) among the three replicate experiments of the healthy donor (HD), anti–double-stranded DNA (anti-dsDNA)^neg^ systemic lupus erythematosus (SLE), and anti-dsDNA^pos^ SLE samples.


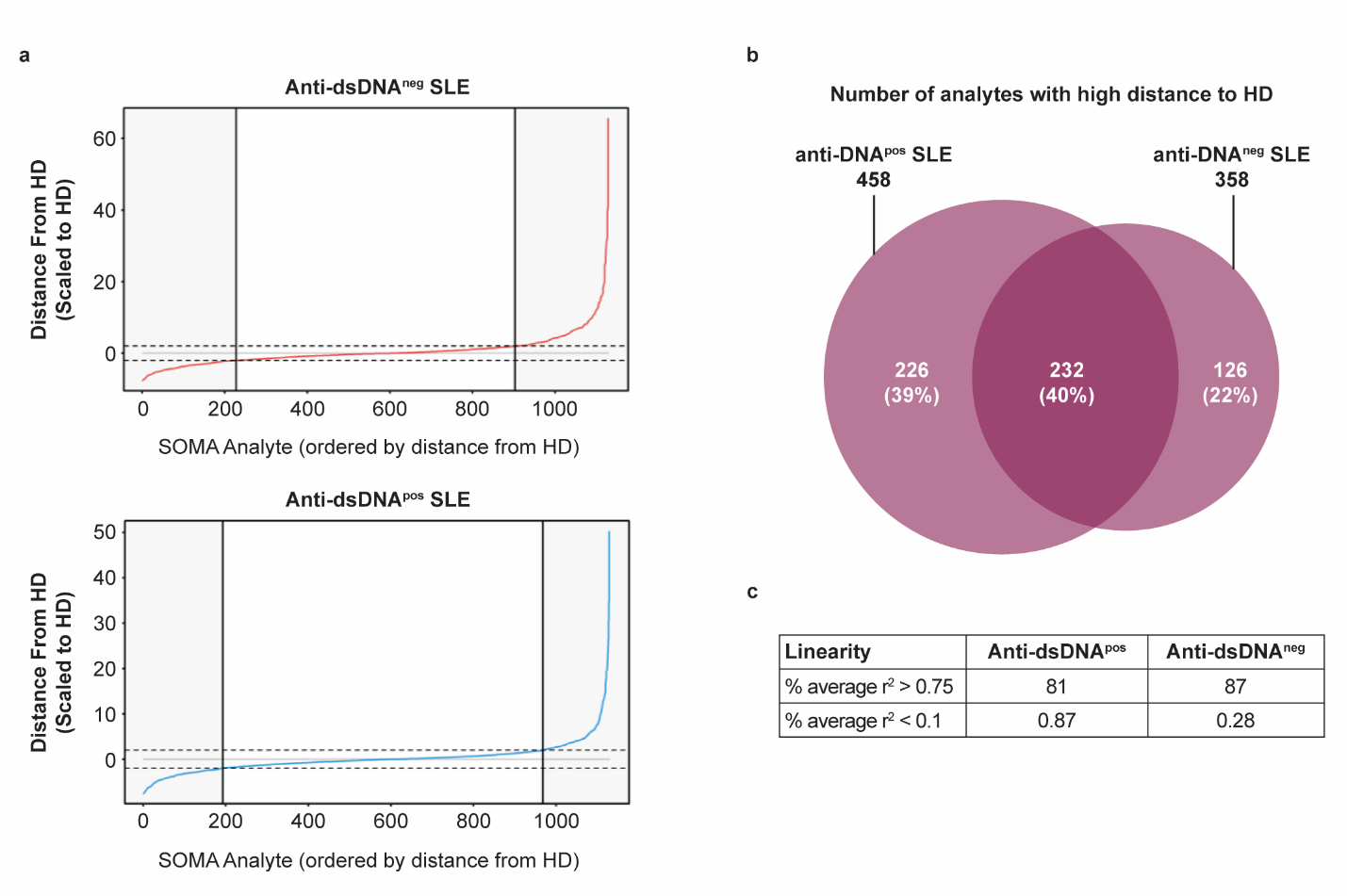


Supplementary Figure S4. (a) 1,129 protein measurements from the 143 systemic lupus erythematosus (SLE) samples and 50 healthy donors (HD) were scaled to the mean and standard deviation of a HD sample. SomaLogic measurements with a distance of greater than 2 standard deviations between either the anti–double-stranded DNA (anti-dsDNA)^pos^ SLE sample or the anti-dsDNA^neg^ SLE sample were assessed based on ability to recover linearity from sample titrations. (b) Venn diagram displaying overlap between markers with high distance from SLE samples to a HD sample. (c) Table displaying percentage of protein measurements that display high linearity (r^2^ > 0.75) and low linearity (r^2^ < 0.1) with dilution.


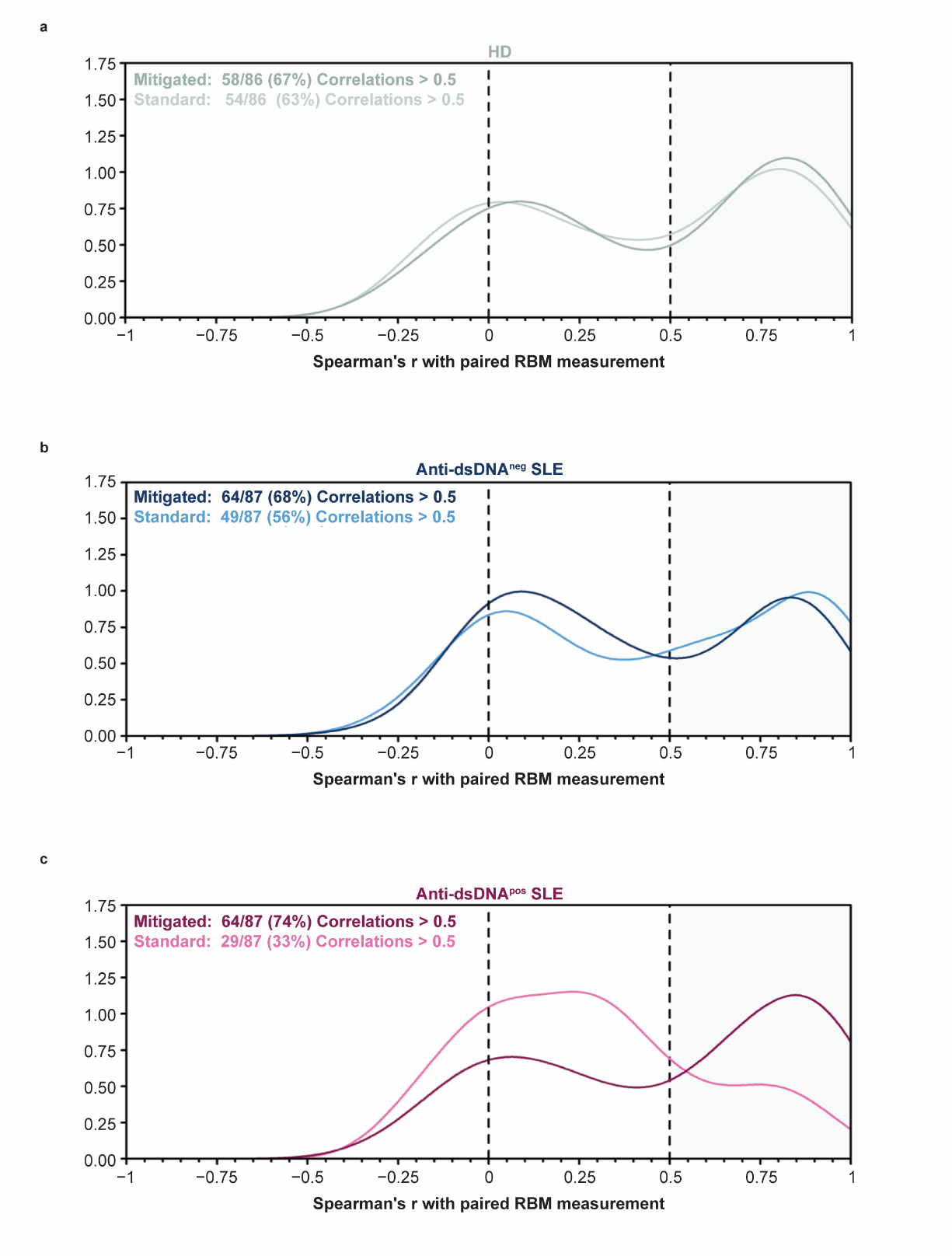


Supplementary Figure S5. Density plots displaying Spearman’s correlation of paired Rules-Based Medicine (RBM) and SomaLogic measurements in the (a) 50 healthy donors (HD), (b) 79 anti–double-stranded DNA (anti-dsDNA)^neg^ systemic lupus erythematosus (SLE), and (c) 64 anti-dsDNA^pos^ SLE samples generated from both the standard and mitigation protocols. Only RBM analytes in which 75% of measurements were above lower limit of quantification in the specific sample group were used in correlation analysis.


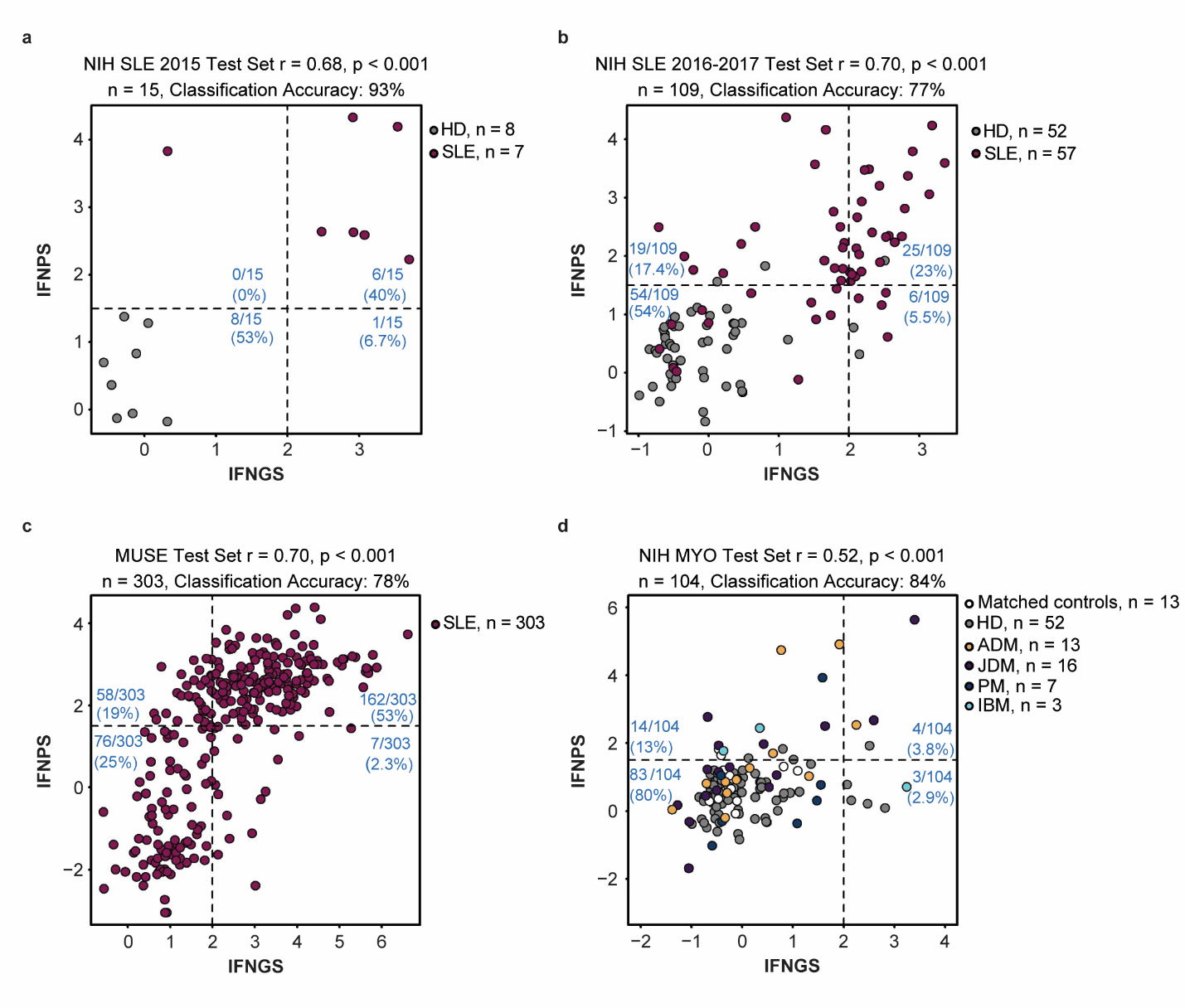


Supplementary Figure S6. Scatterplots displaying concordance between four-protein IFN signature (IFNPS) and the IFN 21-gene signature (IFNGS) in additional test set of samples (a) collected in 2015 from NIH lupus cohort, (b) collected in 2016–2017 from NIH lupus cohort, (c) collected from MUSE cohort at baseline time point, and (d) collected from NIH myositis (MYO) cohort. SLE = systemic lupus erythematosus, HD = healthy donors, ADM = amyopathic dermatomyositis, JDM = juvenile dermatomyositis, PM = polymyositis, IBM = inclusion body myositis.


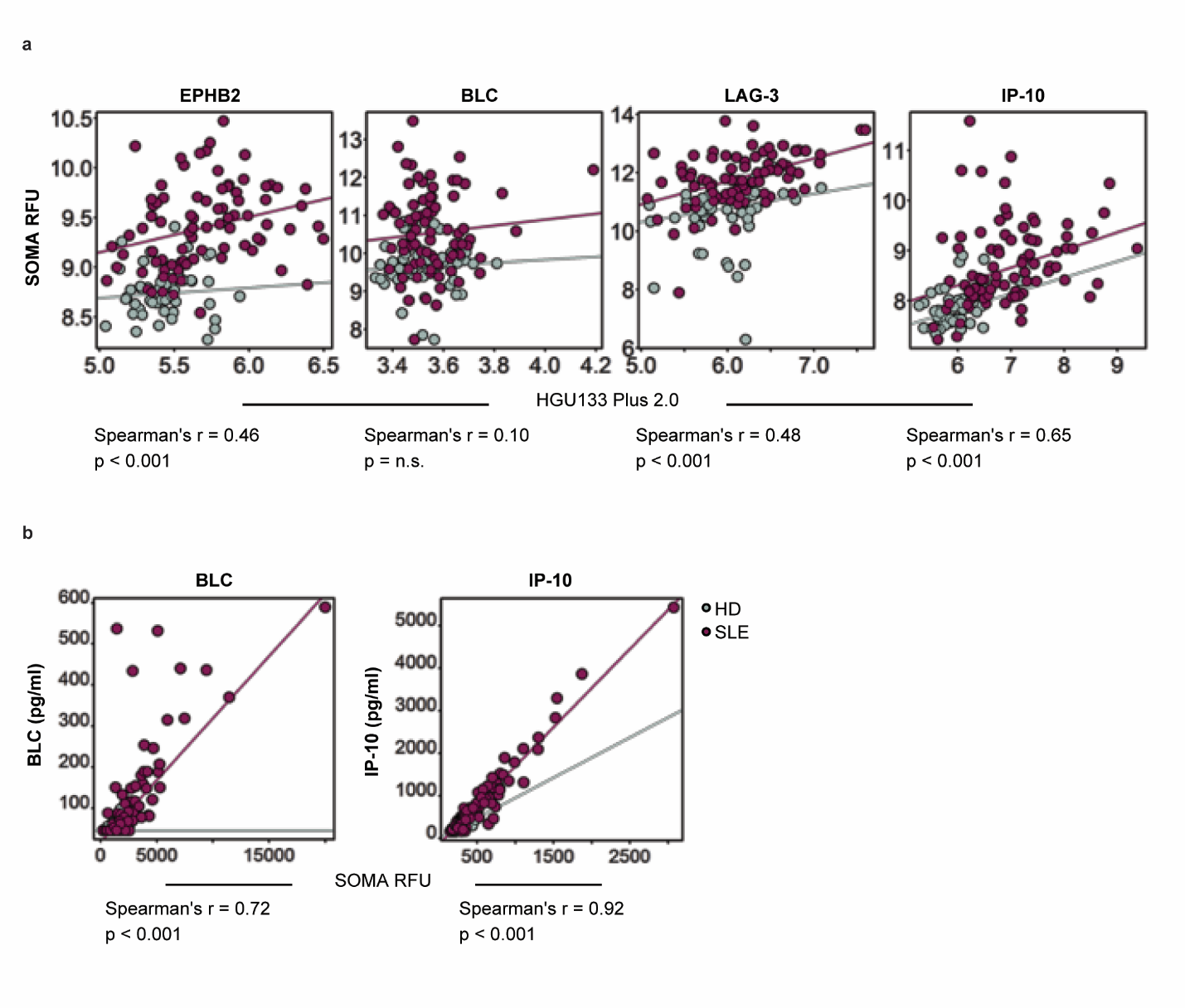


Supplementary Figure S7. Validation of components of SomaLogic measurements of four-protein IFN signature in independent platforms. For each component of the signature, (a) pairwise Spearman’s correlation between the SomaLogic measurements and paired gene expression microarray (Human Genome U133 Plus 2.0 Array) measurements and (b) Rules-Based Medicine (RBM) protein measurements are reported. To visualise correlations between paired SOMAscan and gene expression measurements, a log_2_ transformation was applied to both axes. Scatterplots displaying correlations between paired SomaLogic and RBM protein measurements are scaled linearly on both axes. HD = healthy donors, SLE = systemic lupus erythematosus, RFU = relative fluorescence units.


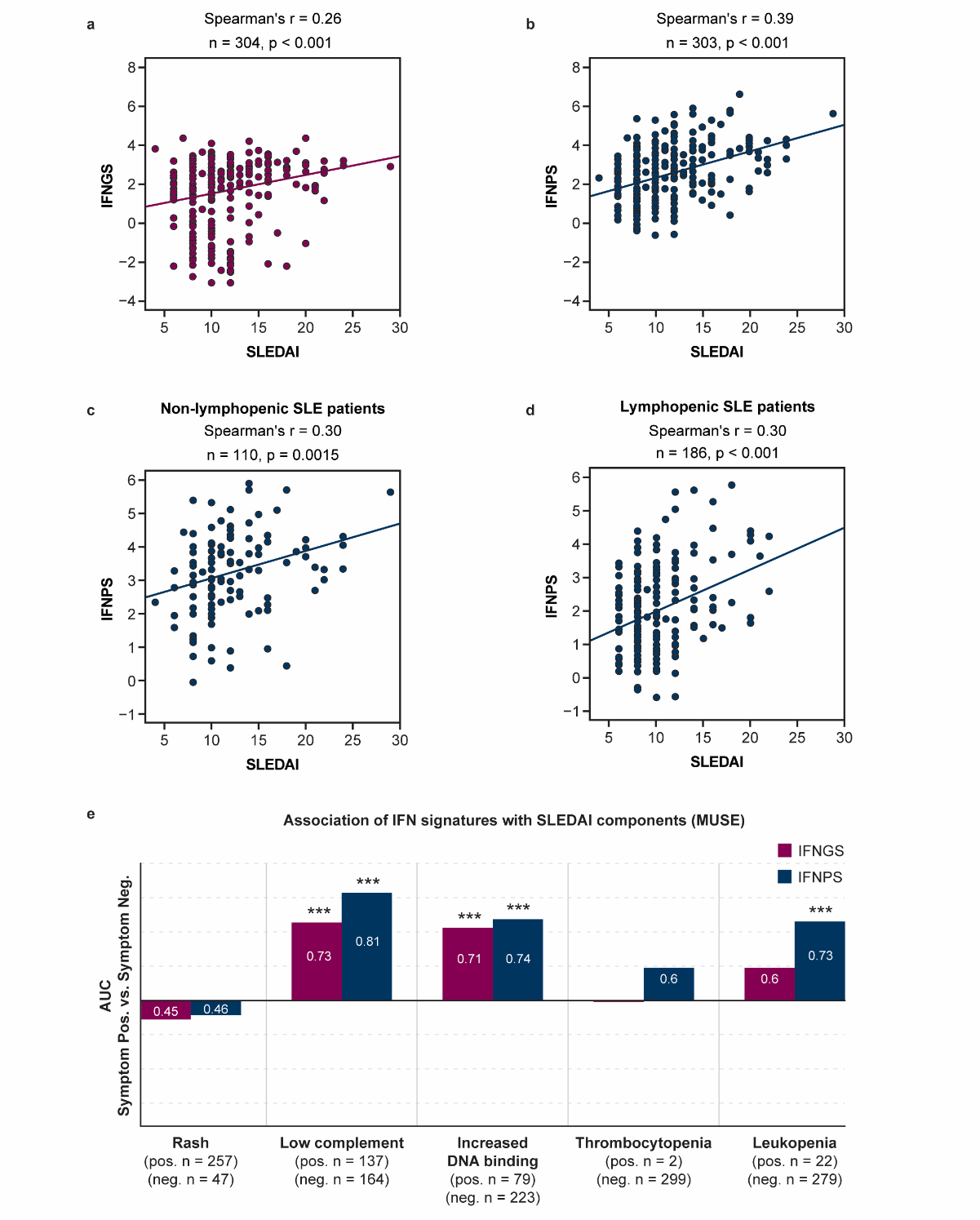


Supplementary Figure S8. Scatterplots displaying correlation between (a) interferon gene signature (IFNGS) and Systemic Lupus Erythematosus Disease Activity Index (SLEDAI) and (b) four-protein IFN signature (IFNPS) and SLEDAI in the MUSE lupus cohort. Scatterplots displaying correlation between IFNPS and SLEDAI in (c) non-lymphopenic patients with systemic lupus erythematosus (SLE) and (d) lymphopenic patients with SLE. (e) Area under the curve (AUC) of IFNGS and IFNPS in discriminating patients with SLE positive or negative for specific SLEDAI components in the MUSE lupus cohort. Threshold for leukopenia < 3,000 WBC/µl. *** p < 0.001, ** p < 0.01, * p < 0.05, ▪ p < 0.10 using Mann-Whitney U test.


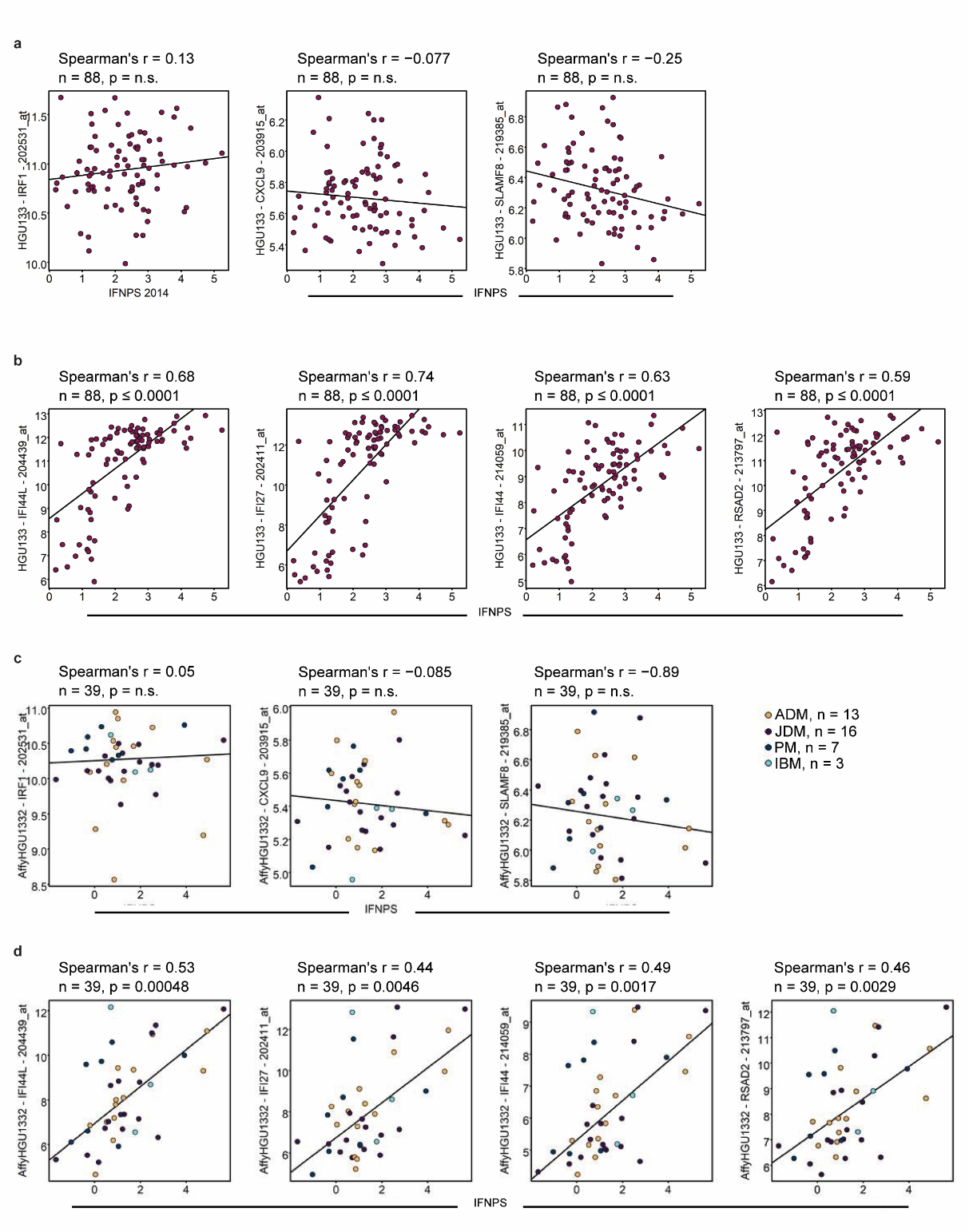


Supplementary Figure S9. Scatterplots displaying correlation between (a) interferon (IFN)-γ–inducible genes IRF1, CXCL9, and SLAMF8 and (b) type I IFN–inducible genes IFI44L, IFI27, IFI44, and RSAD2 and IFN protein signature (IFNPS) in NIH cohort. Scatterplots displaying correlation between (c) IFN-γ–inducible genes IRF1, CXCL9, and SLAMF8 and (d) type I IFN–inducible genes IFI44L, IFI27, IFI44, and RSAD2 and IFNPS in NIH myositis cohort. Line represents best fit from robust regression. n.s. = not significant, ADM = amyopathic dermatomyositis, JDM = juvenile dermatomyositis, PM = polymyositis, IBM = inclusion body myositis.


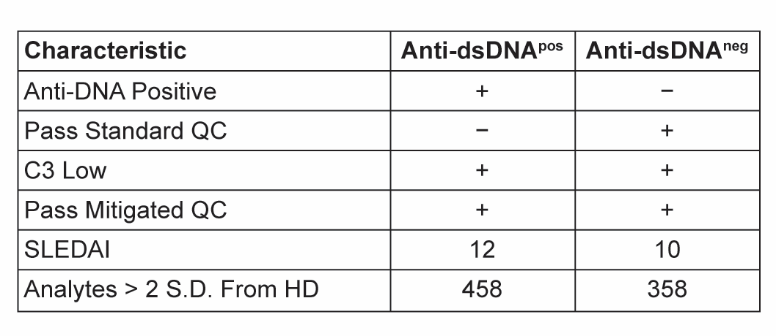


Supplementary Table S1. Characteristics of systemic lupus erythematosus (SLE) samples used in mixed dilution study. Serum from an anti–double-stranded DNA (anti-dsDNA)^pos^ SLE sample and anti-dsDNA^neg^ SLE sample were selected and matched based on low C3, global disease activity as reported by SLE Disease Activity Index (SLEDAI), and the number of analytes > 2 standard deviations (S.D.) from a healthy donor (HD) sample. QC = quality control.


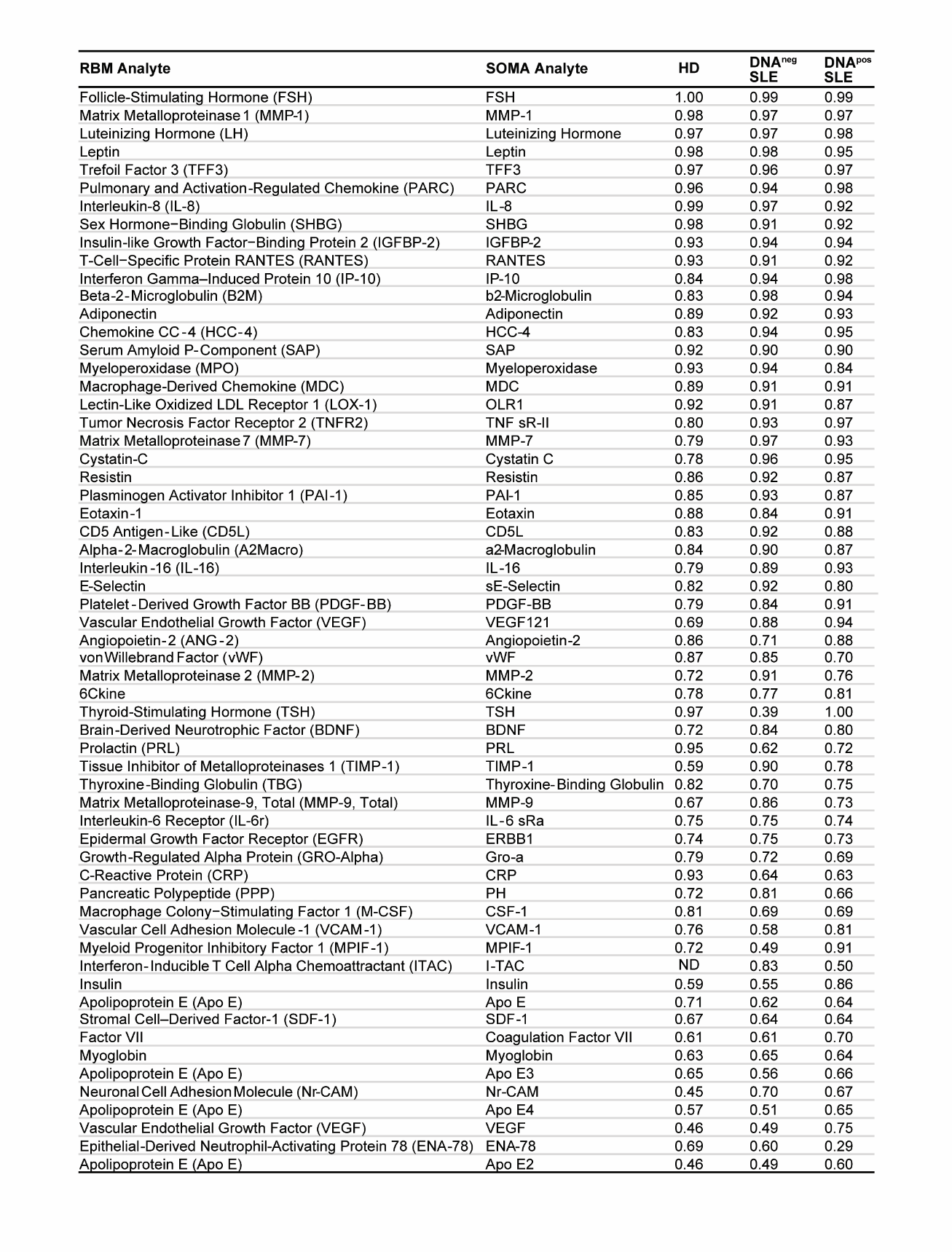


Supplementary Table S2. Spearman’s r between Rules-Based Medicine (RBM) and mitigated SomaLogic measurement in healthy donors (HD), anti–double-stranded DNA (anti-dsDNA)^neg^ systemic lupus erythematosus (SLE), and anti-dsDNA^pos^ SLE samples in which average Spearman’s correlation coefficient was > 0.5 in all three groups. ND = not detectable.


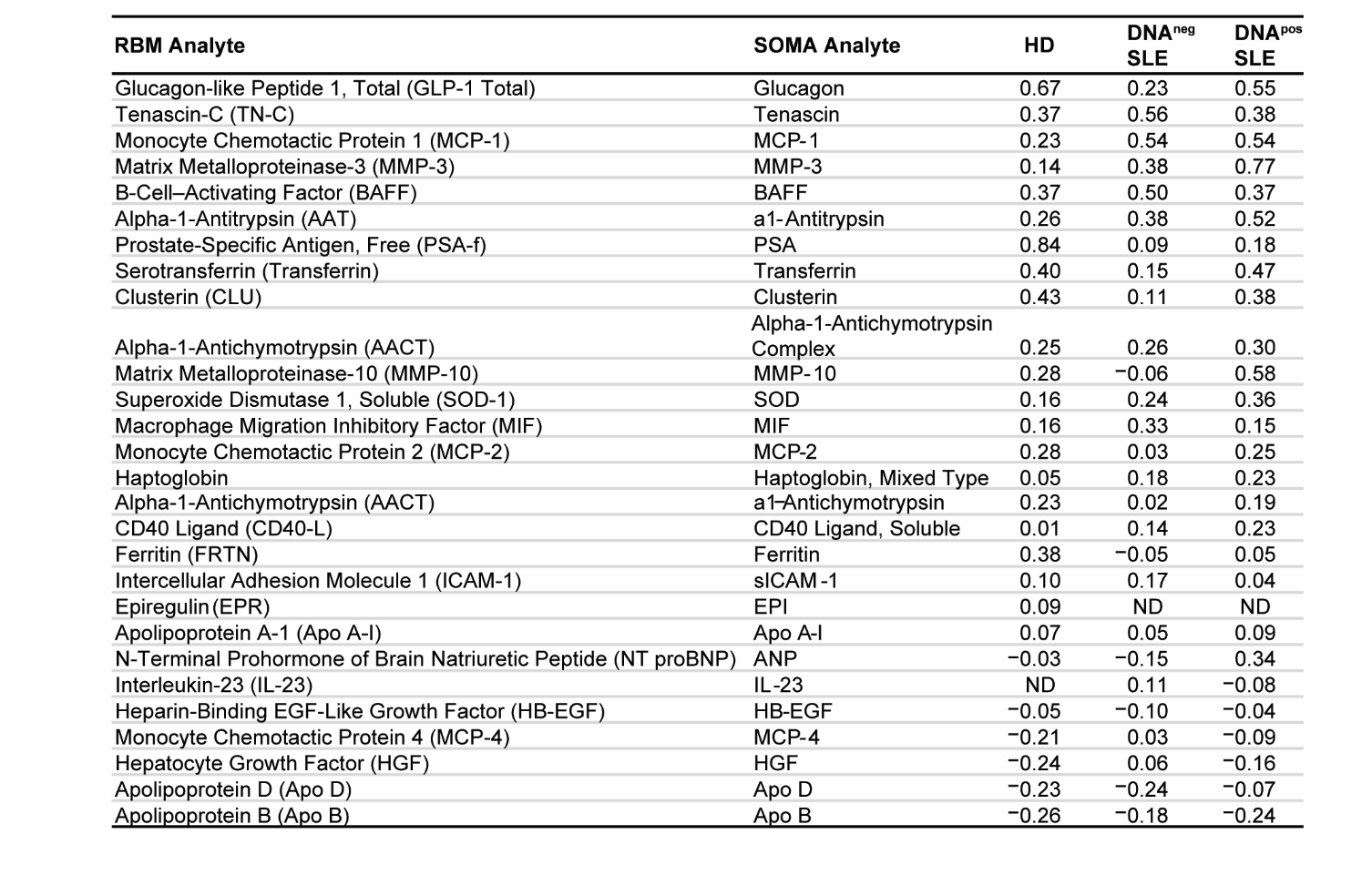


Supplementary Table S3. Spearman’s r between Rules-Based Medicine (RBM) and mitigated SomaLogic measurement in healthy donors (HD), anti–double-stranded DNA (anti-dsDNA)^neg^ systemic lupus erythematosus (SLE), and anti-dsDNA^pos^ SLE samples in which average Spearman’s correlation coefficient was < 0.5 in all three groups. ND = not detectable.


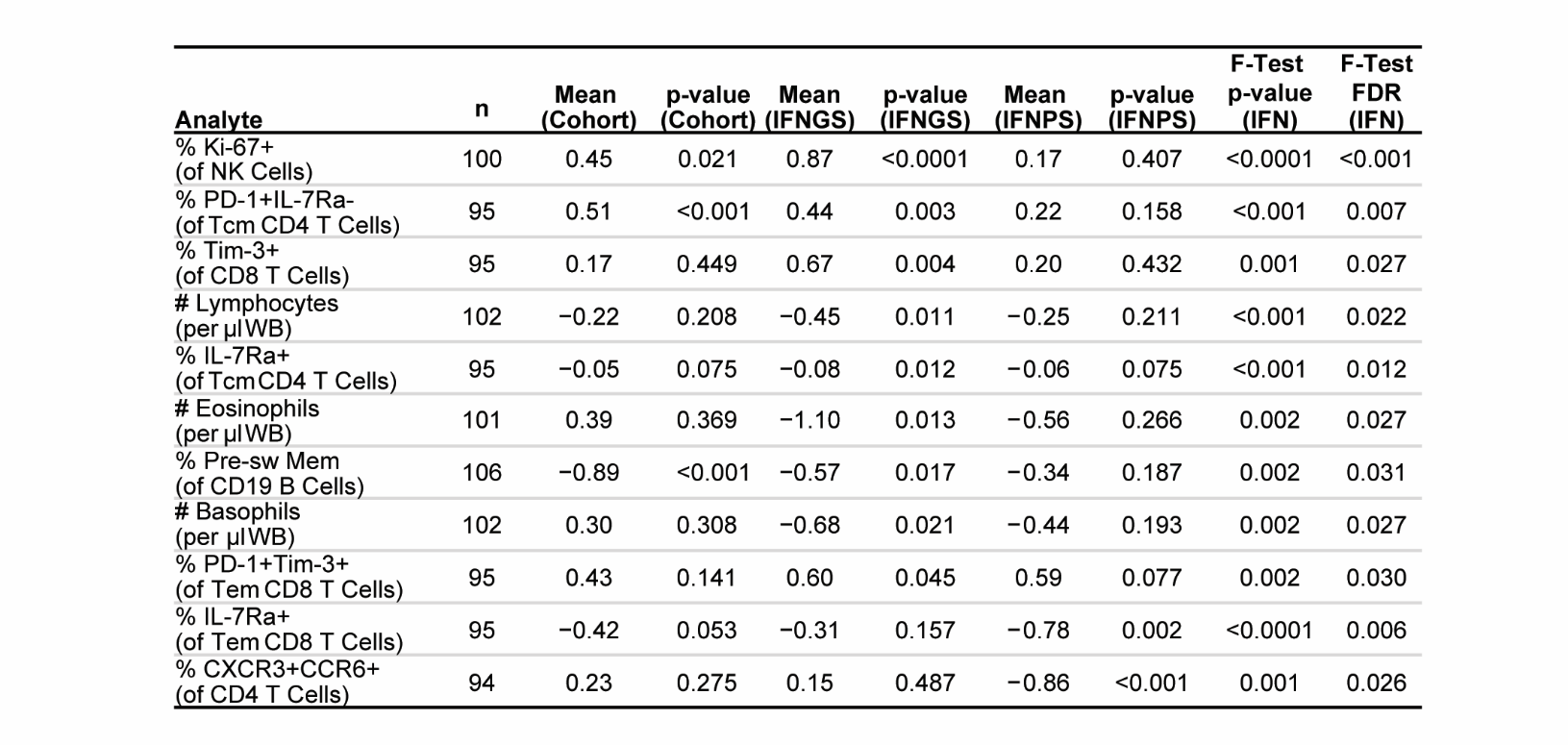


Supplementary Table S4. Cell populations significantly associated with type I interferon (IFN) protein (IFNPS) or gene signature (IFNGS) in multiple regression model fit using each cell population sequentially as dependent variable, treating IFNGS-high/-low, IFNPS-high/-low, and disease status (systemic lupus erythematosus vs. healthy donors) as covariates. An F-test was used to assess statistically significant associations between different cell populations and the IFNPS and IFNGS combined. Cell populations with a false discovery rate (FDR) < 0.05 were considered significant. T-tests were then performed to assess the independent association of each cell population with the IFNGS or IFNPS.


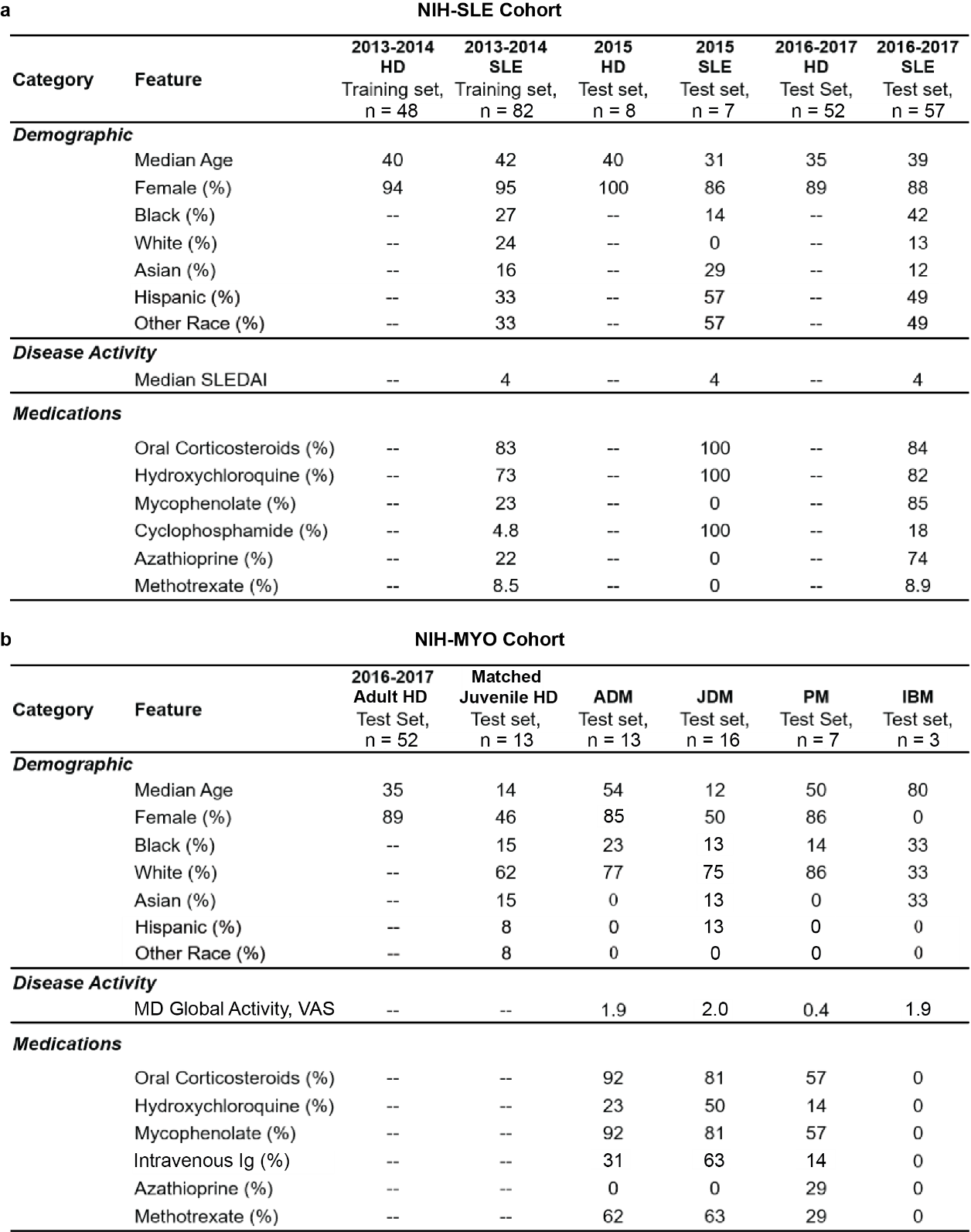


Supplementary Table S5. Demographic and clinical characteristics. HD = healthy donors, MYO = myositis, SLE = systemic lupus erythematosus, SLEDAI = SLE, Disease Activity Index, DAS = disease activity score, ADM = amyopathic dermatomyositis, JDM = juvenile dermatomyositis, PM = polymyositis, IBM = inclusion body myositis.
